# Supplementary material for: Cyclic di-GMP differentially tunes a bacterial flagellar motor through a novel class of CheY-like regulators
Source: eLife. 2017 Nov 1;6:e28842. doi: 10.7554/eLife.28842 (PMC5677366; doi:10.7554/eLife.28842)
Supplement: Supplementary file 1. — (A) Strains used in this study. (B) Plasmids used in this study. (C) Oligonucleotids used in this study. [file elife-28842-supp1.docx]

**Supplementary File 1A: Strains used in this study**

**Name Genotype and description Reference or source**

*E. coli* strains

DH10B F‐*mcrA* Δ(*mrr*‐*hsd* RMS‐ *mcrBC*) φ80d*lacZM*15D*lacX*74 *endA1* rec1deoR D(*ara*, *leu*)7697 *araD*139 *galU* *nupG* *rpsL* *thi* *prohsd*+ *recA* [1]

RP4‐2‐Tc::Mu‐Tn7

S17.1 F‐, lambda (‐), *thi*, *pro*, *recA*, restriction (‐) modification (+),RP4 derivative integrated into the chromosome with Tet::Mu, Km::T7 [1]

BL21 Expression host for pET21 vectors; genomic integrated T7-RNApolymerase under control of lac promoter; deficient in Lon and OmpT Stratagen

(DE3) protease

MG1655 λ^-^, *rph*-1 [2]

AB1770 MG1655 *csrA*::kan::Frt Δ*cyaA*::Frt S. Steiner

*C.crescentus strains*

NA1000 WT; Synchronizable laboratory strain of CB15 (CB15N)

[1,3]

UJ5511 NA1000 hfsA+; NA1000 with functional *hfsA* [4]

UJ5673 NA1000 Δ*cleA*; Markerless in frame deletion of *CC0440* in NA1000 This work

UJ5677 NA1000 ∆*cleB*; Markerless in frame deletion of *CC1364* in NA1000 This work

UJ6650 NA1000 ∆*cleC*; Markerless in frame deletion of *CC2249* in NA1000 This work

UJ5676 NA1000 ∆*cleD*; Markerless in frame deletion of *CC3100* in NA1000 This work

UJ6652 NA1000 ∆*cleE*; Markerless in frame deletion of *CC3155* in NA1000 This work

UJ7853 NA1000 ∆*cleA* to *E*; Markerless in frame deletion of *CC0440*, *CC1364*, *CC2249*, *CC3100* and *CC3155* in NA1000 This work

UJ4467 NA1000 ∆pdeA; Markerless in frame deletion of *pdeA* in NA1000 [1,5]

UJ6804 NA1000 ∆*pdeA* ∆*cleA;* Markerless in frame deletion of *pdeA* in UJ5673 This work

UJ6806 NA1000 ∆*pdeA* ∆*cleB;* Markerless in frame deletion of *pdeA* in UJ5677 This work

UJ6808 NA1000 ∆*pdeA* ∆*cleC;* Markerless in frame deletion of *pdeA* in UJ6650 This work

UJ5832 NA1000 ∆*pdeA* ∆*cleD*, Markerless in frame deletion of *pdeA* in UJ5676 This work

UJ6810 NA1000 ∆*pdeA* ∆*cleE;* Markerless in frame deletion of *pdeA* in UJ6652 This work

UJ8294 NA1000 ∆*pdeA* ∆*cleA to E*; Markerless in frame deletion of *pdeA* in UJ7853 This work

UJ7040 NA1000 Δ*pilA*; Markerless in frame deletion of *pilA* in NA1000 This work

UJ7047 NA1000 Δ*pilA* ∆*cleA*; Markerless in frame deletion of *pilA* in UJ5673 This work

UJ7051 NA1000 Δ*pilA* ∆*cleB*; Markerless in frame deletion of *pilA* in UJ5677 This work

UJ7049 NA1000 Δ*pilA* ∆*cleC*; Markerless in frame deletion of *pilA* in UJ6650 This work

UJ7042 NA1000 Δ*pilA* ∆*cleD*; Markerless in frame deletion of *pilA* UJ5676 This work

UJ7053 NA1000 Δ*pilA* ∆*cleE*; Markerless in frame deletion of *pilA* in UJ6652 This work

UJ8647 NA1000 Δ*pilA* ∆*cleB* to *E*; Markerless in frame deletion of *pilA,* *CC1364, CC2249 CC3100, and CC3155* in NA1000 This work

UJ8648 NA1000 Δ*pilA* ∆*cleA* to *E*; Markerless in frame deletion of *pilA,* *CC0440*, *CC1364, CC2249 CC3100, and CC3155* in NA1000 This work

UJ8028 NA1000 Δ*pilA* ∆*cheYI*; Markerless in frame deletion of *pilA* and *cheYI* This work

UJ8659 NA1000 Δ*pilA* ∆*cheYII*; Markerless in frame deletion of *pilA* and *cheYII* This work

UJ8660 NA1000 Δ*pilA* ∆*cheYI*; Markerless in frame deletion of *pilA* and *cheYI* and *cheYII* This work

UJ8662 NA1000 Δ*pilA* ∆*cleA* ∆*cheYII*; Markerless in frame deletion of *pilA, CC0440* and *cheYII* This work

UJ6353 NA1000 *cleD*-*flag*; Markerless in frame addition of a tripple flag-tag to express it at the C-terminus of CC3100 This work

UJ7751 NA1000 *cleA*-*flag*; Markerless in frame addition of a tripple flag-tag to express it at the C-terminus of CC0440 This work

LS1218 NA1000 ∆*fliF;* Markerless in frame deletion of *fliF* in NA1000

[1,6]

UJ8860 NA1000 ∆*fliF* ∆*cleA*; Markerless in frame deletion of *CC0440* in LS1218 This work

UJ8857 NA1000 ∆*fliF* ∆*cleD*; Markerless in frame deletion of *CC3100* in LS1218 This work

LS2356 NA1000 ∆*fliG*; Markerless in frame deletion of *fliG* in NA1000 [2,7]

UJ8861 NA1000 ∆*fliG* ∆*cleA*; Markerless in frame deletion of *CC0440* in LS2356 This work

UJ8858 NA1000 ∆*fliG* ∆*cleD*; Markerless in frame deletion of *CC3100* in LS2356 This work

CJW1524 NA1000 ∆*fliM*; Markerless in frame deletion of *fliM* in NA1000 [8]

UJ8862 NA1000 ∆*fliM* ∆*cleA;* Markerless in frame deletion of *CC0440* in CJW1524 This work

UJ8859 NA1000 ∆*fliM* ∆*cleD;* Markerless in frame deletion of *CC3100* in CJW1524 This work

UJ8649 NA1000 ∆*fliM* ∆*pilA*; Markerless in frame deletion of *pilA* in CJW1524 This work

MT292 NA1000 Δ*cheB1* Δ*cheB2;* Markerless in frame deletion of *CC0436* and *CC0597* in NA1000 [9]

UJ7037 NA1000 Δ*cheB1* Δ*cheB2 ΔpilA;* Markerless in frame deletion of *pilA*, in MT292 This work

MT293 NA1000 Δ*cheR1* Δ*cheR2* Δ*cheR3 ;* Markerless in frame deletion of *CC0435, CC0598* and *CC3472*  in NA1000 [9]

UJ7039 NA1000 Δ*cheR1* Δ*cheR2* Δ*cheR3 ΔpilA ;* Markerless in frame deletion of *pilA* in MT293 This work

CB15 *C. crescentus* wild type ATCC 19089 *Caulobacter vibrioides* LOT:3967454

UJ4467 CB15 ∆*pdeA*; Markerless in frame deletion of *pdeA* in CB15 [10]

UJ5695 CB15 ∆*cleA*; Markerless in frame deletion of *CC0440* in CB15 This work

UJ5694 CB15 ∆*cleB*; Markerless in frame deletion of *CC1364* in CB15 This work

UJ6654 CB15 ∆*cleC*; Markerless in frame deletion of *CC2249* in CB15 This work

UJ5692 CB15 ∆*cleD*; Markerless in frame deletion of *CC3100* in CB15 This work

UJ6656 CB15 ∆*cleE*; Markerless in frame deletion of *CC3155* in CB15 This work

UJ8810 CB15 ∆*cleC* ∆*cleE*; Markerless in frame deletion of *CC2249* and *CC3155* in CB15 This work

UJ8784 CB15 ∆*cleC* ∆*cleE* ∆*cleA*; Markerless in frame deletion of *CC2249, CC3155 and* *CC0440* in CB15 This work

UJ8790 CB15 ∆*cleC* ∆*cleE* ∆*cleB*; Markerless in frame deletion of *CC2249, CC3155 and* *CC2364* in CB15 This work

UJ8792 CB15 ∆*cleC* ∆*cleE* ∆*cleD*; Markerless in frame deletion of *CC2249, CC3155 and* *CC3100* in CB15 This work

UJ8851 CB15 ∆*cleA* to *E*; Markerless in frame deletion of *CC0440*, *CC1364*, *CC2249*, *CC3100* and *CC3155* in CB15 This work

UJ5831 CB15 ∆*pdeA* ∆*cleD*, Markerless in frame deletion of *CC3100* in UJ4467 This work

UJ9048 NA1000 hfsA+ ∆*fliM*, Markerless in frame deletion of *fliM* in strain NA1000 hfsA+ This work

UJ8849 CB15 ∆*cheYI*, Markerless in frame deletion of *cheYI* in strain CB15 This work

UJ8850 CB15 ∆*cheYII*, Markerless in frame deletion of *cheYII* in strain CB15 This work

UJ776 NA1000 hfsA+ ∆*pilA*, Markerless in frame deletion of pilA in strain NA1000 hfsA+ This work

UJ9632 CB15 ∆*pilA* ∆*cleA-E*, Markerless in frame deletions of *pilA*, *CC0440*, *CC1364*, *CC2249*, *CC3100* and *CC3155* in strain CB15 This work

UJ8177 NA1000 hfsA+ ∆*flgDE*, Markerless in frame deletions of *flgDE* in strain NA1000 hfsA+ This work

UJ8178 NA1000 hfsA+ ∆*flgDE* ∆*pilA*, Markerless in frame deletions of *flgDE* and *pilA* in strain NA1000 hfsA+ This work

UJ9611 CB15 ∆*flgDE* ∆*cleA-E*, Markerless in frame deletions of *flgDE,* *CC0440*, *CC1364*, *CC2249*, *CC3100* and *CC3155* in strain CB15 This work

UJ10089 NA1000 hfsA+ *fliM_ID57WA* This work

UJ7055 NA1000 ∆*dgcB* ∆*pilA*, Markerless in frame deletions of *dgcB* and *pilA* in strain NA1000 This work

UJ8179 NA1000 hfsA+ *motB_D33N* [4]

UJ9064 NA1000 hfsA+ *dgcB_E261Q* [4]

UJ10201 CB15 *fliM_ID57WA* ∆*cleA-E*, Markerless in frame deletions of *fliM_ID57WA,* *CC0440*, *CC1364*, *CC2249*, *CC3100* and *CC3155* in strain CB15 This work

UJ9631 CB15 ∆*flgDE* ∆*motB* ∆*cleA-E*, Markerless in frame deletions of *flgDE, motB,* *CC0440*, *CC1364*, *CC2249*, *CC3100* and *CC3155* in strain CB15 This work

UJ9630 CB15 ∆*flgDE* ∆*dgcB* ∆*cleA-E*, Markerless in frame deletions of *flgDE*, *dgcB,* *CC0440*, *CC1364*, *CC2249*, *CC3100* and *CC3155* in strain CB15 This work

UJ8180 NA1000 hfsA+ *motB_D33N* ∆*pilA*, *motB_D33N* and markerless in frame deletion of *pilA* strain NA1000 hfsA+ This work

1. Evinger M, Agabian N. Envelope-associated nucleoid from Caulobacter crescentus stalked and swarmer cells. Journal of Bacteriology. 1977;132: 294–301.

2. Blattner FR, Plunkett G, Bloch CA, Perna NT. The complete genome sequence of Escherichia coli K-12. …. 1997. doi:10.1126/science.277.5331.1453

3. Muir RE, Gober JW. Regulation of late flagellar gene transcription and cell division by flagellum assembly in Caulobacter crescentus. Molecular Microbiology. 2001;41: 117–130.

4. Hug I, Deshpande S, Sprecher KS, Pfohl T, Jenal U. Second messenger-mediated tactile response by a bacterial rotary motor. Science.

5. Abel S, Bucher T, Nicollier M, Hug I, Kaever V, Abel zur Wiesch P, et al. Bi-modal Distribution of the Second Messenger c-di-GMP Controls Cell Fate and Asymmetry during the Caulobacter Cell Cycle. Viollier PH, editor. PLoS Genetics. 2013;9: e1003744. doi:10.1371/journal.pgen.1003744.s014

6. Grünenfelder B, Gehrig S, Jenal U. Role of the cytoplasmic C terminus of the FliF motor protein in flagellar assembly and rotation. Journal of Bacteriology. 2003;185: 1624–1633.

7. Jenal U, Shapiro L. Cell cycle-controlled proteolysis of a flagellar motor protein that is asymmetrically distributed in the Caulobacter predivisional cell. The EMBO Journal. 1996;15: 2393–2406.

8. Obuchowski PL, Jacobs-Wagner C. PflI, a Protein Involved in Flagellar Positioning in Caulobacter crescentus. Journal of Bacteriology. 2008;190: 1718–1729. doi:10.1128/JB.01706-07

9. Briegel A, Beeby M, Thanbichler M, Jensen GJ. Activated chemoreceptor arrays remain intact and hexagonally packed. Molecular Microbiology. 2011;82: 748–757. doi:10.1111/j.1365-2958.2011.07854.x

10. Abel S, Chien P, Wassmann P, Schirmer T, Kaever V, Laub MT, et al. Regulatory Cohesion of Cell Cycle and Cell Differentiation through Interlinked Phosphorylation and Second Messenger Networks. Molecular Cell. Elsevier Inc; 2011;43: 550–560. doi:10.1016/j.molcel.2011.07.018

**Supplementary File 1B: Plasmids used in this study**

**Name Description^1^ Oligos used for PCR Reference or source**

pNPTS138 Kan^R^, suicide vector containing *sacB* and oriT D. Alley

pNPTS138-ΔCC0440 pNPTS138, HindIII-upstream region of *CC0440*–KpnI–downstream 3376, 3377, 3378, 3379 This study

region of *CC0440*-BamHI

pNPTS138-ΔCC1364 pNPTS138, HindIII-upstream region of *CC1364*–KpnI–downstream 3400, 3401, 3402, 3403 This study

region of *CC1364*-BamHI

pNPTS138-ΔCC2249 pNPTS138, EcoRI-upstream region of *CC2249*–KpnI–downstream 4732, 4733, 4734, 4735 This study

region of *CC2249*-HindIII

pNPTS138-ΔCC3100 pNPTS138, HindIII-upstream region of *CC3100*–BamHI–downstream 3387, 3388, 3389, 3390 This study

region of *CC3100*-EcoRI

pNPTS138-ΔCC3155 pNPTS138, EcoRI-upstream region of *CC3155*–KpnI–downstream 4738, 4739, 4740, 4741 This study

region of *CC3155*-HindIII

pNPTS138-CC3100-flag pNPTS138, HindIII- *CC3100*-*flag*-ClaI-*flag*–downstream region of 3390, 4665, 4666, 4667 This study *CC3100-*EcoRI

pNPTS138-CC0440-flag pNPTS138-HindIII-*CC0440*-*flag*ClaI-*flag*-downstream region of 3379, 6540, 6541, 6542 This study

CC0440-BamHI

pNPTS138-ΔcheYI pNPTS138-BamHI-upstream region of *cheYI* – KpnI- downstream 6734, 6735, 6736, 6737 This study

region of *cheYI* - HindIII

pNPTS138-ΔcheYII pNPTS138-BamHI-upstream region of *cheYII* – KpnI- downstream 6734, 6735, 6736, 6737 This study

region of *cheYII* - HindIII

pNPTSRXH2 pNPTS138-Δ*pilA* Jeff Skerker

pMT687 Kan^R^, pRXMCS_2, low copy replicating plasmid, xylose inducible promoter [1]

pMT687-CleD pCC3100; pMT687, NdeI-*CC3100*-KpnI 3615, 3616 This study

pMT687-CleD-rec pMT687, NdeI-*CC3100* *rec*–KpnI 3615, 5398 This study

pMT687-CleD-rec-ARR pMT687, NdeI-*CC3100* *rec* *arr*-KpnI 3615, 5399 This study

pMT687-CleD_R156A pMT687, NdeI-*CC3100* C466G-KpnI 3615, 3616, 5360, 5361 This study

pMT687-CleD_R169A pMT687, NdeI-*CC3100* CG505GC-KpnI 3615, 3616, 5366, 5367 This study

pMT687-CleD_R113A pMT687, NdeI-*CC3100* CG337GC-KpnI 3615, 3616, 8219, 8220 This study

pMT687-CleD_E70A pMT687, NdeI-*CC3100* A209G-KpnI 3615, 3616, 5621,5622 This study

pMT687-CleD_T101A pMT687, NdeI-*CC3100* A301G-KpnI 3615, 3616, 6714, 6715 This study

pMT687-CleA pMT687, EcoRI-*CC0440*-KpnI 3621, 3622 This study

pMT687-CleA_D67A pMT687, EcoRI-*CC0440* A200C-KpnI (template pMT375-CC0440 D67A) 3621, 3622 This study

pMT687-CleA_R111A pMT687, EcoRI-*CC0440* CG331GC-KpnI 3621, 3622, 8221,8222 This study

pMT687-CleA_RR153AA pMT687, EcoRI-*CC0440* CGACGA457GCAGCA-KpnI 3621, 3622, 6166, 6167 This study

pMT687-CleA_R167A pMT687, EcoRI-*CC0440* AG499GC–KpnI 3621, 3622, 8223, 8224 This study

pSRK-Km Kan^R^, pBBR based medium‐ copy number plasmid, IPTG inducible [2]

pSRK-FliM pSRK-Km, NdeI-fliM-HindIII 5470, 5471 This study

pSRK-FliM_ID57WA pSRK-Km, NdeI-fliM (ATCGAC169GAGTGG)-HindIII 5470, 5471 6545,6546 This study

pSRK-cleA pSRK-Km, NdeI-cleA-HindIII 8638, 8639 This study

pSRK-cleB pSRK-Km, NdeI-cleB-HindIII 8640, 8641 This study

pSRK-cleC pSRK-Km, NdeI-cleC-HindIII 8642, 8643 This study

pSRK-cleD pSRK-Km, NdeI-cleD-HindIII 8644, 8645 This study

pSRK-cleE pSRK-Km, NdeI-cleE-HindIII 8646, 8647 This study

pMT745 CmR, pGFP; pRVGFPC_6, low copy plasmid for C-terminal GFP fusions, [1]

vanillate inducible promoter

pEF84 pMT745, NdeI-*CC3100*-KpnI 4662, 4663 This study

pMT745-CleD-rec pMT745, NdeI-*CC3100* *rec*-KpnI 4662, 5599 This study

pMT745-CleD-rec-ARR pMT745, NdeI-*CC3100* *rec* *arr*-KpnI 4662, 5600 This study

pMT745-CleD_R156A pMT745, NdeI-*CC3100* C466G-KpnI 4662, 4663 This study

(template pMT687-CleD R156A)

pMT745-CleD_R169A pMT745, NdeI-*CC3100* CG505GC-KpnI 4662, 4663 This study

(template pMT687-CleD R169A)

pMT745-CleD_R113A pMT745, NdeI-*CC3100* CG337GC-KpnI 4662, 4663 This study

(template pMT687-CleD R113A)

pMT745-CleD_E70A pMT745, NdeI-*CC3100* A209G-KpnI 4662, 4663 This study

(template pMT687-CleD E70A)

pDHL580 Amp^R^, Kan^R^, pUC19-linker-mGFPmut3-FRT-*kan*-FRT [3]

pMT375 TetR, pMCS-5 [1]

pMT375-mGFPmut3 pMT375; KpnI-*mgfpmut3*-NheI (template pDHL580) 5331, 5332 This study

pMT375-CleA pMT375; EcoRI-*CC0440*-KpnI 3621, 5229 This study

pMT375-CleA-mGFPmut3 pMT375-CC0440; KpnI-*mgfpmut*3-NheI (template pDHL580) 5331, 5332 This study

pMT375-CleA_D67A pMT375, EcoRI-*CC0440* A200C-KpnI 3621, 5477, 5476,5229 This study

pMT375-CleA_D67A-mGFPmut3 pMT375 CleA D67A, KpnI-*mGFPmut3*-NheI (template pDHL580) 5331, 5332 This study

pMT375-CleA_RR153AA-mGFPmut3 pMT375-mGFPmut3, EcoRI-*CC0440* CGACGA457GCAGCA-KpnI 3621, 5229 This study

(template pMT687-CleA RR153AA)

pMT375-CleA_R111A-mGFPmut3 pMT375-mGFPmut3, EcoRI-*CC0440* CG331GC-KpnI 3621, 5229 This study

(template pMT687-CleA R111A)

pMT375-CleA_R167A-mGFPmut3 pMT375-mGFPmut3, EcoRI-*CC0440* AG499GC-KpnI 3621, 5229 This study

(template pMT687-CleA R167A)

pET28a Kan^R^, expression vector, T7 promoter Novagen

pET28a-His-CleD pET28a-NdeI-*CC3100*-HindIII 3291, 3615 This study

pET28a-His-CleD (aa 19-269) pET28a, NdeI-*CC3100* (corresponding aa 19 -269) )-HindIII 3100Fwd, 3100 Fl Rvs This study

pET28a-His-CleD-rec (aa 19-141) pET28a, NdeI-*CC3100* *rec* (corresponding aa 19-141)-HindIII 3100Fwd, 3100 RecRvs This study

pET28a-His-CleD-rec ARR (aa 19-174) pET28a, NdeI-*CC3100* *rec arr* (corresponding aa 19–174)-HindIII 3100Fwd, 3100 RecArgRvs This study

pET28a-His-CleD -GFPmut1 pET28a-*CC3100*-*gfpmut1* (RF cloning; templates

pET22b-His-GFPmut1 and pET28a-His-CC3100) 3100GFP-Fwd, 3100GFP-RVS This study

pET28-His-CleD_R113A-GFPmut1 pET28a-*CC3100* CG337GC-*gfpmut*1 (mutagenesis; 3100 R113A Fwd,

template pET28a-His-CleD GFPmut1) 3100 R113Rvs This study

pET28a-His-EcCheY pET28a, *Nde*I-Ec*cheY*-*Hin*dIII (template MG1655) 6722, 6723 This study

pET28a-His-EcCheY-CleD (ARR) pET28a, *Nde*I-Ec*cheY*-*arr* (*CC3100*)-EcoRI 6722, 6724, 6725, 6741 This study

(template MG1655 and NA1000)

pET28a-His-EcCheY-CleD (ARR_R156A) pET28a, *Nde*I-Ec*cheY*-*arr* (CC3100 C466G)-EcoRI 6722, 6724, 6725, 6741 This study

(template MG1655 and pMT687-CleD R156A)

pET28a-His-SUMO pET28a-His-SUMO

[4]

pET28a-His-SUMO-ARR-CTR_CleA_ pET28a-His-*arr* *CC0440*; RF cloning; templates SUMO ARR0440 Fwd

pET28a-His-SUMO, pMT687-CleA) SUMO ARR0440 Rvs This study

pET28a-His-SUMO-ARR_CleB_ pET28a-His-*arr* (*CC1364* RF cloning; templates SUMO ARR1364 Fwd

pET28a-His-SUMO, NA1000) SUMO ARR1364 Rvs This study

pET28a-His-SUMO-ARR_CleC_ pET28a-His-*arr* (*CC2249*; RF cloning; templates SUMO ARR2249 Fwd

pET28a-His-SUMO, NA1000) SUMO ARR2249 Rvs This study

pET28a-His-SUMO-ARR_CleD_ pET28a-His-*arr* (CC3100; RF cloning; templates SUMO ARR3100 Fwd

pET28a-His-SUMO, pET28a-His-CleD) SUMO ARR3100 Rvs This study

pET28a-His-SUMO-ARR_CleD__R156A pET28a-His-*arr* (CC3100; RF cloning; templates SUMO ARR3100 Fwd

pET28a-His-SUMO, pMT687-CleD R156A) SUMO ARR3100 Rvs This study

pET28a-His-SUMO-ARR_CleD_ _R169A pET28a-His-*arr* (CC3100; RF cloning; templates SUMO ARR3100 Fwd

pET28a-His-SUMO, pMT687-CleD R169A) SUMO ARR3100 Rvs This study

pET28a-His-SUMO- ARR_CleE_ pET28a-His-*arr* (CC3155; RF cloning; templates SUMO ARR3155 Fwd

pET28a-His-SUMO, NA1000) SUMO ARR3155 Rvs This study

pET28-StrepII-CleD pET28a, NcoI-*strepII*-BamHI-*CC3100-*HindIII 3287, 3288, 3290, 3291 This study

pET22b-His-GFPmut1 Amp^R^, pET22b to express proteins with N-terminal His and C-terminal GFP tag S. Hiller

pKT25 Kan^R^, pSU40 derivative, used for fusions to the C-terminus of the T25 [5]

fragment of CyaA

pKT25-zip pKT25; region coding for the leucine zipper of GCN4 3` fused to the [5]

T25 fragment

pKT25-CleA pKT25-BamHI-*CC0440*-EcoRI 6732, 6733 This study

pKT25-CleD pKT25-BamHI-*CC3100*-EcoRI 4438, 4439 This study

pKT25-FliM (aa 2-65) pKT25-BamHI-*fliM* (corresponding to aa 2-65)-EcoRI 6726/6727 This study

pKT25-FliM_ID57WA (aa 2-65) pKT25-BamHI-*fliM* ATCGAC169GAGTGG (corresponding to aa 2-65)-EcoRI 6726/6727 This study

(template pSRK-FliM ID_WA)

pUT18 Amp^R^, pUC19 derivative, used for fusions to the T18 fragment of CyaA, which is [5]

in the fusion protein N-terminal:.

pUT18-FliM (aa 2-65) pUT18-BamHI-*fliM* (corresponding to aa 2-65)-EcoRI 6726/6862 This study

pUT18-FliM_ID57WA (aa 2-65) pUT18-BamHI- *fliM* ATCGAC169GAGTGG (corresponding to aa 2-65)-EcoRI 6726/6862 This study

(template pSRK-FliM ID_WA)

pUT18-CleD pUT18-BamHI-CC3100-EcoRI 4438, 4439 This study

pUT18C Amp^R^, pUC19 derivative, used for fusions to the T18 fragment of CyaA, which is [5]

in the fusion protein C-terminal

pUT18C-zip pUT18C; region coding for the leucine zipper of GCN4 3` fused to the [5]

T18 fragment

pUT18C-FliM (aa 2-65) pUT18C-BamHI-*fliM* (corresponding to aa 2-65)-EcoRI 6726/6727 This study

pUT18C-FliM_ID57WA (aa 2-65) pUT18C-BamHI- *fliM* ATCGAC169GAGTGG (corresponding to aa 2-65)-EcoRI 6726/6727 This study

(template pSRK-FliM_ID57WA)

pUT18C-CleA pUT18C-BamHI-*CC0440*-EcoRI 6732, 6733 This study

pUT18C-CleD pUT18C-BamHI-*CC3100*-EcoRI 4438, 4439 This study

^1^When not noted otherwise chromosomal DNA of NA1000 was used as template for PCR.

1. Thanbichler M, Iniesta AA, Shapiro L. A comprehensive set of plasmids for vanillate- and xylose-inducible gene expression in Caulobacter crescentus. Nucleic Acids Research. 2007;35: e137–e137. doi:10.1093/nar/gkm818

2. Khan SR, Gaines J, Roop RM, Farrand SK. Broad-Host-Range Expression Vectors with Tightly Regulated Promoters and Their Use To Examine the Influence of TraR and TraM Expression on Ti Plasmid Quorum Sensing. Applied and Environmental Microbiology. 2008;74: 5053–5062. doi:10.1128/AEM.01098-08

3. Landgraf D, Okumus B, Chien P, Baker TA, Paulsson J. Segregation of molecules at cell division reveals native protein localization. Nature Methods. 2012;9: 480–482. doi:10.1038/nmeth.1955

4. Fumeaux C, Radhakrishnan SK, Ardissone S, Théraulaz L, Frandi A, Martins D, et al. Cell cycle transition from S-phase to G1 in Caulobacter is mediated by ancestral virulence regulators. Nature Communications. 2014;5: 4081. doi:10.1038/ncomms5081

5. Karimova G, Pidoux J, Ullmann A, Ladant D. A bacterial two-hybrid system based on a reconstituted signal transduction pathway. Proceedings of the National Academy of Sciences of the United States of America. 1998;95: 5752–5756.

**Supplementary File 1C: Oligonucleotids used in this study**

**Name Sequence^1^ Description**

3287 ATATACCATGGGATGGAGCCACCCGCAGTTCGAAAAAGGATCCAAGCTT cloning of StrepII tag in pET28a

3288 AAGCTTGGATCCTTTTTCGAACTGCGGGTGGCTCCATCCCATGGTATAT cloning of StrepII tag in pET28a

3290 AAGGATCCTTCGTGTTTGACGGCAACGT cloning of *CC3100* together with StrepII tag in pET28a

3291 CCGCAAGCTTCTAGGCCGCGCGCCCCCGGT cloning of *CC3100* together with StrepII tag in pET28a

3376 ATATAAGCTTCCTTCGCCGAACGTTTCCTG cloning of the upstream region of *CC0440* in pNPTS138

3377 GGGGTACCGGTCGAAGGAAGGAGAGCGT cloning of the upstream region of *CC0440* in pNPTS138

3378 GGGGTACCTGACCGTCCGCAAATTTAAA cloning of the downstream region of *CC0440* in pNPTS138

3379 CGGGATCCTGGCCTTGAAGTCTTCGTTT cloning of the downstream region of *CC0440* in pNPTS138

3387 ATATAAGCTTATGGTCGAGGCCTTGCTG cloning of the upstream region of *CC3100* in pNPTS138

3388 TTGGATCCGTTGCCGTCAAACACGAACA cloning of the upstream region of *CC3100* in pNPTS138

3389 TTGGATCCTAGAGCATTTTCCGATCTGT cloning of the downstream region of *CC3100* in pNPTS138

3390 TCAGAATTCCTCGATGGGCGGCTATGT cloning of the downstream region of *CC3100* in pNPTS138

3400 TACTAAGCTTGGTTTCGACCTTTGACAACA cloning of the upstream region of *CC1364* in pNPTS138

3401 AAGGTACCCACATCGCGGAAATCCATTC cloning of the upstream region of *CC1364* in pNPTS138

3402 AAGGTACCGAAGACATCTACTAGGCCCG cloning of the downstream region of *CC1364* in pNPTS138

3403 TTGGATCCCTGCTGCCGTTCCTGTTC cloning of the downstream region of *CC1364* in pNPTS138

3407 AAGCTGTTCGACGGCCTGTC screening of *CC0440* deletion mutants

3408 CTTGGCCTTCGTCTCGGCTC screening of *CC0440* deletion mutants

3413 ATGAAAGTCGGCGAGGACAG screening of *CC1364* deletion mutants

3414 GTTTGACCTGTCCAACCCCT screening of *CC1364* deletion mutants

3415 ACATGGGCGGGGGCGGAGCG screening of *CC3100* deletion mutants

3416 ACCTTCGCCCTCGCCGGCTT screening of *CC3100* deletion mutants

3615 GCGGCAGCCATATGTTCGTGTTTGACGGCAACGT cloning of *CC3100* and point mutants into pMT687

3616 GTGGTACCCTAGGCCGCGCGCCCCCGGT cloning of *CC3100* and point mutants into pMT687

3621 ATGAATTCAACAGACGCTCTCCTTCCTTC cloning of *CC0440* and point mutants into pMT687

3622 GTGGTACCTCATATCTGCACCTTCGCTG cloning of *CC0440* and point mutants into pMT687

4438 TAGAGGATCCGTTCGTGTTTGACGGCAACGT cloning of *CC3100* in pKT25, pUT18 and pUT18C

4439 AAGAATTCCTAGGCCGCGCGCCCCCGGT cloning of *CC3100* in pKT25, pUT18 and pUT18C

4662 AAAGCATATGTTCGTGTTTGACGGCAAC cloning of *CC3100* in pMT745

4663 TTTAGGTACCGGCCGCGCGCCCCCGGTC cloning of *CC3100* in pMT745

4665 tttgtaATCGATatcatgatctttataatcaccgtcatggtctttgtagtcggccgcgcgcccccggtcctc cloning of CC3100-*flag* in pNPTS138

4666 aaagatATCGATtacaaggatgacgatgacaagtagAGCATTTTCCGATCTGTTTGGATTGG cloning of *CC3100*-*flag* in pNPTS138

4667 ttttgctagcCTGGATACCTCGATCCATGTC cloning of *CC3100*-*flag* in pNPTS138

4732 TAGAATTCATGGCTCCGCTGTGGACGAA cloning of the upstream region of *CC2249* in pNPTS138

4733 GTGGTACCCTTTTCGAGCACCGCCGACA cloning of the upstream region of *CC2249* in pNPTS138

4734 TAGGTACCACCCTCGAAATCTAGCTGGA cloning of the downstream region of *CC2249* in pNPTS138

4735 GTTCCAAGCTTCGCCAGCCGCAATCTGAAAC cloning of the downstream region of *CC2249* in pNPTS138

4736 GATGGCTGGTTCGTCATCGG screening of *CC2249* deletion mutants

4737 GTGCGCATGATGGGTGTGGA screening of *CC2249* deletion mutants

4738 GTGAATTCTCGAAAAGATCCGCAAGGCC cloning of the upstream region of *CC3155* in pNPTS138

4739 TTGGTACCCTTCAGGAGGTCGAACCGCA cloning of the upstream region of *CC3155* in pNPTS138

4740 TAGGTACCGACACCGAACGCGTCCATCC cloning of the downstream region of *CC3155* in pNPTS138

4741 TGACAAGCTTTGCTGAGTCTCTAGGATTGG cloning of the downstream region of *CC3155* in pNPTS138

4742 GGGTTGTGATTTCTTCGCGG screening of *CC3155* deletion mutants

4743 GCCTCTATTTCGCTGAGCGC screening of *CC3155* deletion mutants

5229 CTGGTACCTATCTGCACCTTCGCTGGGC cloning of *CC0440* derivatives in pMT375

5331 GATAGGTACCGGTGGCGGTGGCAGTAAAGG cloning of mGFPmut3 in pMT375-*CC0440* derivatives

5332 GGCTGCAGCTAGCTTATTTGTAGAGTTCATCCA cloning of mGFPmut3 in pMT375-*CC0440* derivatives

5360 CGGGCCTGATCGCGCTCGTTTCAACTC cloning of CC3100 R156A in pMT687

5361 GAGTTGAAACGAGCGCGATCAGGCCCG cloning of CC3100 R156A in pMT687

5366 AGGGGCCGCGCAAGGCCAAGGCGGACGCCA cloning of CC3100 R169A in pMT687

5367 TGGCGTCCGCCTTGGCCTTGCGCGGCCCCT cloning of CC3100 R169A in pMT687

5398 GAGGTACCTTACGGCTTCAGCGTCACCGCCT cloning of the rec domain of CC3100 in pMT687

5399 GAGGTACCTTAGCTGGCGTCCGCCTTGCGCT cloning of the rec domain and ARR of CC3100 in pMT687

5470 GACGACCATATGGGCGGCTGGGATGACGG cloning of *fliM* in pSRK-Km

5471 CCGGTACCTTTCAGTTTCTGCACGGCCT cloning of *fliM* in pSRK-Km

5476 CGATCTGATCATCAGCGCCGTCCAGATGCCGGTCAC introduction of point mutations (D67A) in CC0440

5477 GTGACCGGCATCTGGACGGCGCTGATGATCAGATCG introduction of point mutations (D67A) in CC0440

5599 GAGGTACCCGGCTTCAGCGTCACCGCCT cloning of the rec domain of CC3100 in pMT745

5600 GAGGTACCGCTGGCGTCCGCCTTGCGCT cloning of the rec domain and ARR of CC3100 in pMT745

5621 AGCTGATCTTCGTCGCGCACGGTAGCAGCGG cloning of CC3100 E70A in pMT687

5622 CCGCTGCTACCGTGCGCGACGAAGATCAGCT cloning of CC3100 E70A in pMT687

6166 TTCGTCGGGCCGGATGCAGCATTCAAGCATATGGGCC introduction of point mutations (RR153AA) in CC0440

6167 GGCCCATATGCTTGAATGCTGCATCCGGCCCGACGAA introduction of point mutations (RR153AA) in CC0440

6540 TCATGATATCGATTACAAGGATGACGATGACAAGTGACCGTCCGCAAATTTAAA cloning of *CC0440-flag* in pNPTS138

6541 ATCCTTGTAATCGATATCATGATCTTTATAATCACCGTCATGGTCTTTGTAGTCTATCTGCACCTTCGCTGGGC cloning of *CC0440-flag* in pNPTS138

6542 GCAAAAGCTTACGCTCTCCTTCCTTCGACC cloning of *CC0440-flag* in pNPTS138

6545 CATTCTGAACCAGGACGAGTGGGCCAGCCTGCTGGGCTTCGAT cloning of FliM (ID-WA) in pSRK-Km

6546 ATCGAAGCCCAGCAGGCTGGCCCACTCGTCCTGGTTCAGAATG cloning of FiM (ID-WA) in pSRK-Km

6714 GTCATCATGTGCGCCAGCGAAGCCA cloning of CC3100 T101A in pMT687

6715 TGGCTTCGCTGGCGCACATGATGAC cloning of CC3100 T101A in pMT687

6722 GCGGCAGCCATATGGCGGATAAAGAACTTAA cloning of Ec *cheY* in pET28a

6723 CCGCAAGCTTTCACATGCCCAGTTTCTCAA cloning of Ec *cheY* in pET28a

6724 CCATTCGCGCGGCTTCTCAAAGATTTTGTTGAGTT or cloning of Ec *cheY-*ARR *(CC3100)* in pET28a

6725 AACAAAATCTTTGAGAAGCCGCGCGAATGGGTCGA cloning of Ec *cheY-*ARR *(CC3100)* in pET28a

6726 CTAGAGGATCCAGCGGACGAACTCGACGATCA cloning of FliM (corresponding to aa 1-66) in pKT25 and pUT18C

6727 CCGAATTCTTAGAGATCGAAGCCCAGCAGGC cloning of FliM (corresponding to aa 1-66) in pKT25 and pUT18C

6732 CTAGAGGATCCAACAGACGCTCTCCTTCCTTC cloning of *CC0440* in pKT25 and pUT18C

6733 CCGAATTCTCATATCTGCACCTTCGCTG cloning of *CC0440* in pKT25 and pUT18C

6734 GAAAGGATCCGCAGGTCAGCCAAGGCGTGA cloning of the upstream region of *cheYI* in pNPTS138

6735 GTTTGGTACCCGTGAGAACCGTACGCGTCA cloning of the upstream region of *cheYI* in pNPTS138

6736 GTTTGGTACCATCGTCAAGCCGTTCAACCC cloning of the downstream region of *cheYI* in pNPTS138

6737 GTTTAAGCTTCTTCGACCGTGATCGTCTGC cloning of the downstream region of *cheYI* in pNPTS138

6738 TCGTCGCTCAGGAAGTCCGG screening of *cheYI* deletion mutants

6739 GAGCGCGGTTTCGTTCGCCT screening of *cheYI* deletion mutants

6741 CCGAATTCTCAGGCGGCGACGCGGCGGA cloning of Ec *cheY-*ARR *(CC3100)* in pET28a

6862 CTGAATTCGCGAGATCGAAGCCCAGCAGGC cloning of *fliM* (corresponding to aa 1-66) in pUT18

7803 GAggatccCAAGGACTTCATGCCCTCGG cloning of the upstream region of *cheYII* in pNPTS138

7804 GAggtaccATGGTGCTTGCTTGGGGCAT cloning of the upstream region of *cheYII* in pNPTS138

7805 GAggtaccTGTTCGGGCAGCTGACATGA cloning of the downstream region of *cheYII* in pNPTS138

7806 GACAGaagcttAGATCCACGTCGCCGCTC cloning of the downstream region of *cheYII* in pNPTS138

7807 TCGTCGCGAAGGTGAAGATC screening of *cheYII* deletion mutants

7808 TCGCAGTCCTGACAGATGG screening of *cheYII* deletion mutants

8219 CTTCGGCGCGgcgGACGCCGGCGTGCAC cloning of *CC3100 R113A* in pMT687

8220 GTGCACGCCGGCGTCcgcCGCGCCGAAG cloning of *CC3100 R113A* in pMT687

8221 GGACTTCGCAGATCGTCAAGATCgcgGACGCCGGCGCCAAC cloning of *CC0440 R111A* in pMT687

8222 GTTGGCGCCGGCGTCcgcGATCTTGACGATCTGCGAAGTCC cloning of *CC0440 R111A* in pMT687

8223 CCGGGGACGGATGGCgcgCGCAAGGATGACCTTCCGG cloning of *CC0440 R167A* in pMT687

8224 CCGGAAGGTCATCCTTGCGcgcGCCATCCGTCCCCGG cloning of *CC0440 R167A* in pMT687

8638 ACCTCAATCATATGACAGACGCTCTCCTTCCTTCG cloning of *CC0440* in pSRK

8639 ACTAACAAGCTTTCATATCTGCACCTTCGCTGGG cloning of *CC0440* in pSRK

8640 ACCTCAATCATATGGATTTCCGCGATGTGTCG cloning of *CC1364* in pSRK

8641 ACTAACAAGCTTCTAGTAGATGTCTTCGAGGTCCATCTCTTC cloning of *CC1364* in pSRK

8642 ACCTCAATCATATGTCGGCGGTGCTCGAAAAG cloning of *CC2249* in pSRK

8643 ACTAACAAGCTTCTAGATTTCGAGGGTCGGGCC cloning of *CC2249* in pSRK

8644 ACCTCAATCATATGTTCGTGTTTGACGGCAACG cloning of *CC3100* in pSRK

8645 ACTAACAAGCTTCTAGGCCGCGCGCCCC cloning of *CC3100* in pSRK

8646 ACCTCAATCATATGCGGTTCGACCTCCTGAAG cloning of *CC3155* in pSRK

8647 ACTAACAAGCTTTTACATTCTCCGCGTCGGATG cloning of *CC3155* in pSRK

3100-REC-Rvs TTTAAGCTTCTACGGCTTCAGCGTCACCGCCTC cloning of the rec domain of CC3100 in pET28a

3100-REC-Arg-Rvs TTTAAGCTTCTAGCTGGCGTCCGCCTTGCGCTT cloning of the rec domain and ARR of CC3100 in pET28a

3100-FL-Rvs TTTAAGCTTCTAAGACCCCATGAACCCCATCAG cloning of *CC3100* in pET28a

3100GFP-Fwd AGGACCGGGGGCGCGCGGCCAGCAAAGGAGAAGAACTTTTCAC restriction free cloning of pET28-His-CC3100-GFPmut1

3100GFP-Rvs AGTGCGGCCGCAAGCTTCTACTTTTTGTAGAGCTCATCCATGC restriction free cloning of pET28-His-CC3100-GFPmut1

3100R113A-Fwd CGCCTCGAGGGCTCGTTCCAGGTCCTTCAG mutagenesis to get pET28-His-CC3100 R113A-GFPmut1

3100R113A-Rvs CTGAAGGACCTGGAACGAGCCCTCGAGGCG mutagenesis to get pET28-His-CC3100 R113A-GFPmut1

SUMO-ARR0440-Fwd TCACCGCGAACAGATTGGTGGCAGTCGAGCGTTCATCGAA restriction free cloning of pET28-His-SUMO-ARR CC0440

SUMO-ARR0440-Rvs GGTGGTGGTGGTGCTCGAGTCACCACCTGCCATCCGTCCC restriction free cloning of pET28-His-SUMO-ARR CC0440

SUMO-ARR1364-Fwd TCACCGCGAACAGATTGGTGGCAGTCGCCCCTTCGTGCGC restriction free cloning of pET28-His-SUMO-ARR CC1364

SUMO-ARR1364-Rvs GGTGGTGGTGGTGCTCGAGTCACCAGCGGCGCTCGACGCC restriction free cloning of pET28-His-SUMO-ARR CC1364

SUMO-ARR2249-Fwd TCACCGCGAACAGATTGGTGGCAGTCGCCCGTTCATCCGG restriction free cloning of pET28-His-SUMO-ARR CC2249

SUMO-ARR2249-Rvs GGTGGTGGTGGTGCTCGAGTCACCAGCGACGGAACGGACC restriction free cloning of pET28-His-SUMO-ARR CC2249

SUMO-ARR3100-Fwd TCACCGCGAACAGATTGGTGGCAGTCGCGAATGGGTCGAG restriction free cloning of pET28-His-SUMO-ARR CC3100

SUMO-ARR3100-Rvs GGTGGTGGTGGTGCTCGAGTCAGCGCTTGCGCGGCCCCTT restriction free cloning of pET28-His-SUMO-ARR CC3100

SUMO-ARR3155-Fwd TCACCGCGAACAGATTGGTGGCAGTCGCCCCTTCATCCGG restriction free cloning of pET28-His-SUMO-ARR CC3155

SUMO-ARR3155-Rvs GGTGGTGGTGGTGCTCGAGTCACCAGCGTCGCCGGGGTCC restriction free cloning of pET28-His-SUMO-ARR CC3100

^1^ Restriction sites are underlined
