# Supplementary material for: Cyclic di-GMP differentially tunes a bacterial flagellar motor through a novel class of CheY-like regulators
Source: eLife. 2017 Nov 1;6:e28842. doi: 10.7554/eLife.28842 (PMC5677366; doi:10.7554/eLife.28842)
Supplement: Transparent reporting form [file elife-28842-transrepform.pdf]

eLIFE
1st Floor
24 Hills Road
Cambridge CB2 1JP, UK
P 01223 855340
W elifesciences.org
T @elife

eLife's transparent reporting form

We encourage authors to provide detailed information *within their submission* to facilitate the interpretation and replication of experiments. If you have any questions, please contact us: editorial@elifesciences.org.

Sample-size estimation

- You should state whether an appropriate sample size was computed when the study was being designed
- You should state the statistical method of sample size computation and any required assumptions
- If no explicit power analysis was used, you should describe how you decided what sample (replicate) size (number) to use

Please outline where this information can be found within the submission (e.g., page numbers or figure legends), or explain why this information doesn't apply to your submission:

For all single cell experiments (motility assays) shown in **Figs. 7, 8, S5, S7, S8** and **S10**, data were collected from more than 850 cells for each strain. Sample sizes are listed in the legends of supplementary figures S5, S7, S8 and S10 and in Materials and Methods chapter on "high speed video tracking and analysis".

Replicates

- You should report how often each experiment was performed
- You should include a definition of biological versus technical replication
- The data obtained should be provided and sufficient information should be provided to indicate the number of independent biological and/or technical replicates
- If you encountered any outliers, you should describe how these were handled
- Criteria for exclusion/inclusion of data should be clearly stated
- High-throughput sequence data should be uploaded before submission, with a private link for reviewers provided (these are available from both GEO and ArrayExpress)

Please outline where this information can be found within the submission (e.g., page numbers or figure legends), or explain why this information doesn't apply to your submission:

Fig.2a-c: 3 technical replicates; **2d**: 2 technical replicates; representative examples are shown.

Fig.3a,b,d,e: 2 technical replicates; **3c**: 3 technical replicates; representative examples are shown.

Fig.4a: 4 biological replicates for strains ΔcheYI, ΔcheYII, ΔYI-YII, fliMID57WA. 8 biological replicates for all other strains. **4b**: 7 biological replicates; **4c**: 8 biological replicates. **4e**: the following number of microcolonies were analyzed for each strain: WT (60), ΔfliM (39), ΔpilA (58), ΔcleA (20), ΔcleB (20), ΔcleC (20), ΔcleD (74), ΔcleE (67), ΔcleA-E (47), ΔcleCE (38), ΔcleABCD (47), ΔcleABDE (82), ΔcleA-E +cleC (46), fliMID57WA (34), ΔcheYI (30), ΔcheYII (45). **4g**: each dot represents a single cell measurement

Fig.5c: numbers of cells analyzed for each strain (top to bottom): 3452, 1450, 637, 2357, 2603, 573, 690, 795, 1148, 1347. **5d**: numbers of cells analyzed for each strain (top to bottom): 226, >200, 287, 2194, >200, >200, 645, >200, >200, >200, 981, 709.

Fig.6a: 3 biological replicates, representative example is shown. **6b**: two technical replicates; **6c**: 3 biological replicates.

Fig.S2a,b,c: 2 technological replicas, representative example shown.

Fig.S4a: 3 biological replicas with standard deviations indicated.
Fig.S4b: 2 biological replicas, representative example shown.

Fig.S5b: 6 biological replicas with standard deviations indicated.

Statistical reporting

- Statistical analysis methods should be described and justified
- Raw data should be presented in figures whenever informative to do so (typically when N per group is less than 10)
- For each experiment, you should identify the statistical tests used, exact values of N, definitions of center, methods of multiple test correction, and dispersion and precision measures (e.g., mean, median, SD, SEM, confidence intervals; and, for the major substantive results, a measure of effect size (e.g., Pearson's r, Cohen's d)
- Report exact p-values wherever possible alongside the summary statistics and 95% confidence intervals. These should be reported for all key questions and not only when the p-value is less than 0.05.

Please outline where this information can be found within the submission (e.g., page numbers or figure legends), or explain why this information doesn't apply to your submission:

For all single cell experiments (motility assays) shown in **Figs. 7, 8, S5, S7, S8** and **S10** n values and definitions of center are indicated in the supplementary figure legends (S8 and S10).

(For large datasets, or papers with a very large number of statistical tests, you may upload a single table file with tests, Ns, etc., with reference to page numbers in the manuscript.)

Additional data files ("source data")

- We encourage you to upload relevant additional data files, such as numerical data that are represented as a graph in a figure, or as a summary table
- Where provided, these should be in the most useful format, and they can be uploaded as "Source data" files linked to a main figure or table
- Include model definition files including the full list of parameters used
- Include code used for data analysis (e.g., R, MatLab)
- Avoid stating that data files are "available upon request"

Please indicate the figures or tables for which source data files have been provided:

The methods for data analysis are written in the Material and Methods section (High speed video tracking and analysis).
